# Supplementary material for: Arabidopsis shoot stem cells display dynamic transcription and DNA methylation patterns
Source: EMBO J. 2020 Aug 20;39(20):e103667. doi: 10.15252/embj.2019103667 (PMC7560203; doi:10.15252/embj.2019103667)
Supplement: Supplementary file 1 — Appendix [file EMBJ-39-e103667-s001.pdf]

**Gutzat et al. Appendix**

**Table of contents**

- **Supplementary Text**
- **References for supplementary Text**
- **Appendix Figures S1-S8**
- **Appendix Table S1**

**Supplementary Text**

**Description of additional genes expressed specifically in stem cells**

Among the 32 genes that were more highly expressed in stem cell nuclei in at least three of the four stages but had no obvious connection to epigenetic regulation, nine are transcription factors (TFs, p-value for enrichment: 8.54e-07), and four of these belong to the plant-specific B3 family of transcription factors. Only one of them, Reproductive Meristem 1 (REM1) was previously described as being expressed in the central zone of vegetative meristems (except the L1 layer), in the central zone of reproductive meristems, and in carpel anlagen (Franco-Zorrilla, Cubas et al., 2002). FD, a basic leucine zipper (bZIP)-containing transcription factor, also known to be expressed in meristems, interacts with Flowering Locus T (FT) to promote flowering (Abe, Kobayashi et al., 2005). Other stem cell-prominent TFs include BHL8/PNF, KNAT6, and the essential shoot meristem identity factor STM, all of which are members of the TALE family. BHL8/PNF is involved in repressing lateral organ boundary genes to maintain meristem function (Khan, Ragni et al., 2015), and KNAT6 contributes to SAM maintenance (Belles-Boix, Hamant et al., 2006). WRKY74, a member of the WRKY transcription factor family, has not yet been characterized in detail. ZPR3 is a small leucine zipper protein, which (together with its functional homolog, ZPR4) negatively regulates HD-ZIP III TFs and controls meristem size (Kim, Kim et al., 2008). Besides the TF genes, we found several genes with largely unknown functions: A serine/threonine kinase; a protein with a development and cell death (DCD) domain; a L11 ribosomal protein family member; a protein with a cysteine-rich domain (PLAC8 family) and a short hypothetical protein with no distinguishing features (At5g03885). For the F-box family protein UFO, our data confirm elevated expression in stem cells of embryos, 7-d and 14-d-old plants, but not in stem cells of inflorescence meristems of 35-d-old plants, consistent with published results (Samach, Klenz et al., 1999). One of the 32 genes is the F-box protein, SAP (*STERILE APETALA*), required for proper growth control (Wang, Li et

al., 2016). Among the shared stem cell DEGs we also found three receptor-like kinases, CLV1, ERL1, and ERL2. CLV1 functions as a receptor for, and its expression overlaps with CLV3 (Trotochaud, Jeong et al., 2000); (Clark, Williams et al., 1997). ERL1 and ERL2 control cell proliferation (Shpak, Berthiaume et al., 2004) by regulating auxin transport (Chen, Wilson et al., 2013).

MCT1 and TEL2 (terminal ear1-like) belong to a plant-specific protein family, with conserved RNA binding motifs (Gu, Jung et al., 2016), and for the latter, a role in meiocytes and gametogenesis has been reported (Anderson, Alvarez et al., 2004, Kaur, Sebastian et al., 2006). A DEG present in all four stages is the cytochrome p450 gene KLU, which plays a role in organ size determination, likely by generating a mobile growth signal (Anastasiou, Kenz et al., 2007). In addition to protein-coding genes, we found one natural antisense RNA overlapping with a gene potentially encoding DRB4B, a dynamin-related protein, and one other noncoding RNA with unknown function.

Up-regulation of these genes in SAM stem cells at all or most developmental stages implies they are functional at these time points and of interest for future functional analyses.

**References for Supplementary Text**


- Abe M, Kobayashi Y, Yamamoto S, Daimon Y, Yamaguchi A, Ikeda Y, Ichinoki H, Notaguchi M, Goto K, Araki T (2005) FD, a bZIP protein mediating signals from the floral pathway integrator FT at the shoot apex. Science 309: 1052-1056
- Anastasiou E, Kenz S, Gerstung M, MacLean D, Timmer J, Fleck C, Lenhard M (2007) Control of plant organ size by KLUH/CYP78A5-dependent intercellular signaling. Developmental Cell 13: 843-856
- Anderson GH, Alvarez NDG, Gilman C, Jeffares DC, Trainor VCW, Hanson MR, Veit B (2004) Diversification of genes encoding mei2-like RNA binding proteins in plants. Plant Molecular Biology 54: 653-670
- Belles-Boix E, Hamant O, Witiak SM, Morin H, Traas J, Pautot V (2006) KNAT6: an Arabidopsis homeobox gene involved in meristem activity and organ separation. Plant Cell 18: 1900-7
- Chen MK, Wilson RL, Palme K, Ditengou FA, Shpak ED (2013) ERECTA family genes regulate auxin transport in the shoot apical meristem and forming leaf primordia. Plant Physiology 162: 1978-1991

- Clark SE, Williams RW, Meyerowitz EM (1997) The CLAVATA1 gene encodes a putative receptor kinase that controls shoot and floral meristem size in Arabidopsis. Cell 89: 575-85
- Franco-Zorrilla JM, Cubas P, Jarillo JA, Fernandez-Calvin B, Salinas J, Martinez-Zapater JM (2002) AtREM1, a member of a new family of B3 domain-containing genes, is preferentially expressed in reproductive meristems. Plant Physiology 128: 418-427
- Gu L, Jung HJ, Kwak KJ, Dinh SN, Kim YO, Kang H (2016) An RRM-containing mei2-like MCT1 plays a negative role in the seed germination and seedling growth of Arabidopsis thaliana in the presence of ABA. Plant Physiol Biochem 109: 273-279
- Kaur J, Sebastian J, Siddiqi I (2006) The Arabidopsis-mei2-like genes play a role in meiosis and vegetative growth in Arabidopsis. Plant Cell 18: 545-59
- Khan M, Ragni L, Tabb P, Salasini BC, Chatfield S, Datla R, Lock J, Kuai X, Despres C, Proveniers M, Cao YG, Xiang DQ, Morin H, Rulliere JP, Citerne S, Hepworth SR, Pautot V (2015) Repression of lateral organ boundary genes by PENNYWISE and POUND-FOOLISH is essential for meristem maintenance and flowering in Arabidopsis. Plant Physiology 169: 2166-2186
- Kim YS, Kim SG, Lee M, Lee I, Park HY, Seo PJ, Jung JH, Kwon EJ, Suh SW, Paek KH, Park CM (2008) HD-ZIP III activity is modulated by competitive inhibitors via a feedback loop in Arabidopsis shoot apical meristem development. Plant Cell 20: 920-933
- Samach A, Klenz JE, Kohalmi SE, Risseeuw E, Haughn GW, Crosby WL (1999) The UNUSUAL FLORAL ORGANS gene of Arabidopsis thaliana is an F-box protein required for normal patterning and growth in the floral meristem. Plant Journal 4: 433-445
- Shpak ED, Berthiaume CT, Hill EJ, Torii KU (2004) Synergistic interaction of three ERECTA-family receptor-like kinases controls Arabidopsis organ growth and flower development by promoting cell proliferation. Development 131: 1491-1501
- Trotochaud AE, Jeong S, Clark SE (2000) CLAVATA3, a multimeric ligand for the CLAVATA1 receptor-kinase. Science 289: 613-617
- Wang Z, Li N, Jiang S, Gonzalez N, Huang X, Wang Y, Inze D, Li Y (2016) SCF(SAP) controls organ size by targeting PPD proteins for degradation in Arabidopsis thaliana. Nat Commun 7: 11192

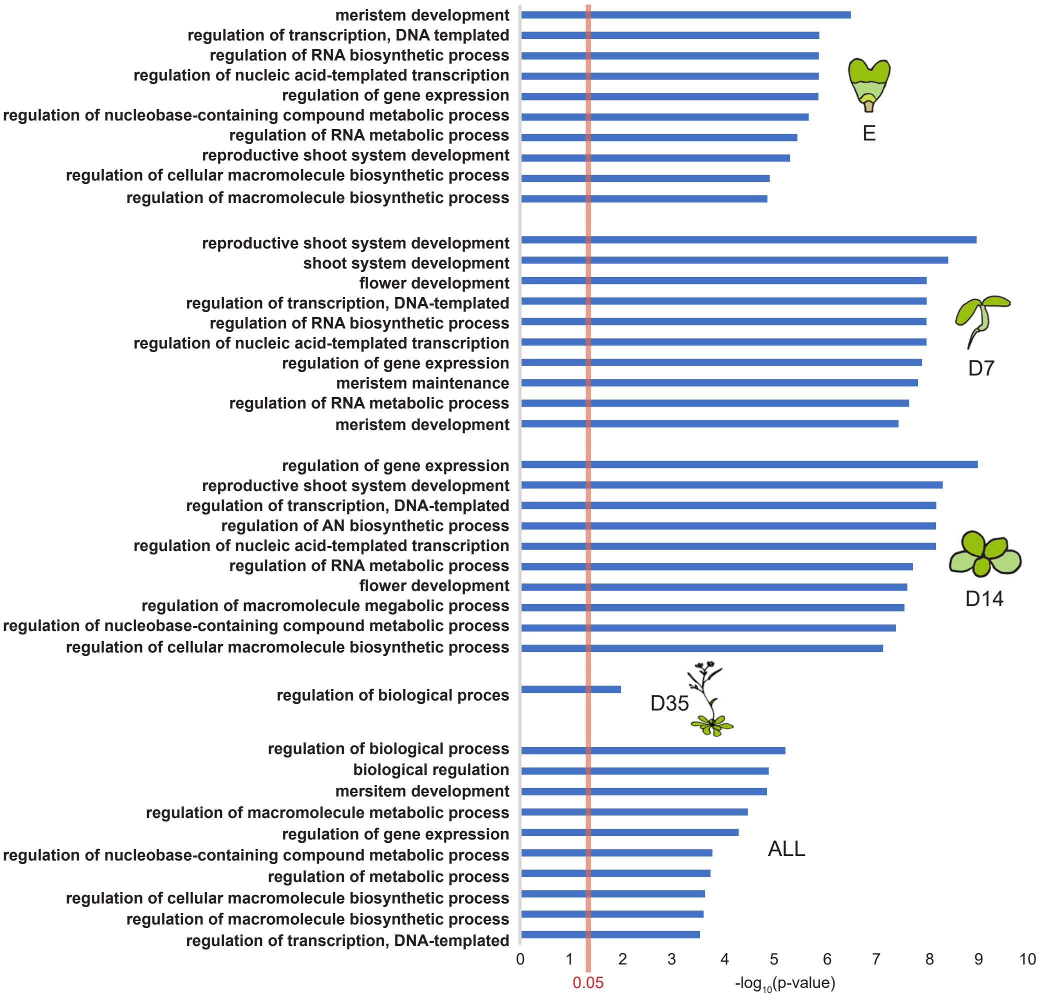


**Appendix Figure S1 | GO-term enrichment.**
Bar plot representing the negative log of the Bonferroni corrected p-value for enrichment of each GO-term in stem cells at the indicated developmental stages. p-values were calculated with Fisher's exact test. A p-value of 0.05 is highlighted by the red line. See also Table EV3 for exact values. s = stem cells; n = non-stem cells.

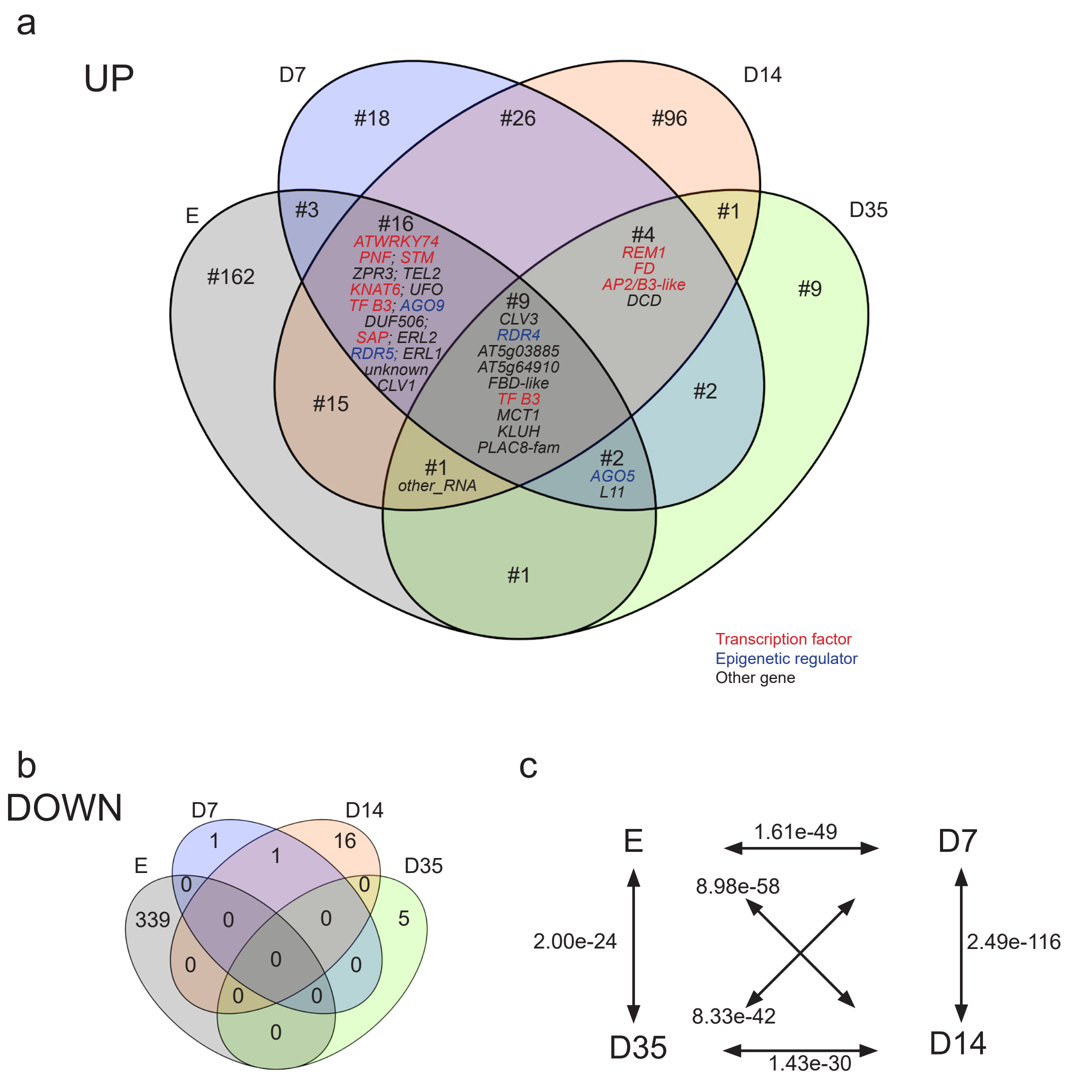


**Appendix Figure S2 | Overlap of DEGs at different timepoints.** (**a**) Venn diagrams for genes up- and (**b**) downregulated in stem cells, respectively. (**c**) p-values (hypergeometric tests) for likelyhood of overlap of upregulated genes in different pairs of timepoints. E = nuclei from embryos, D7/14/35 = nuclei from 7/14/35 day-old plants.

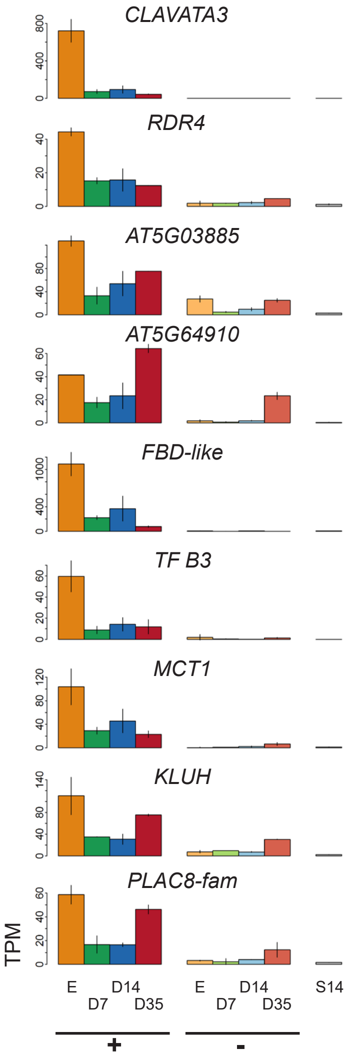


**Appendix Figure S3 | Expression of core stem cell-specific genes.** Bar plots of TPM values of genes that are significantly upregulated in SAM stem cells throughout development. + = stem cells; - = non stem cells, E = nuclei from embryos, D7/14/35 = nuclei from 7/14/35 day-old plants, S14 = nuclei from 14 d-old above-ground seedlings.

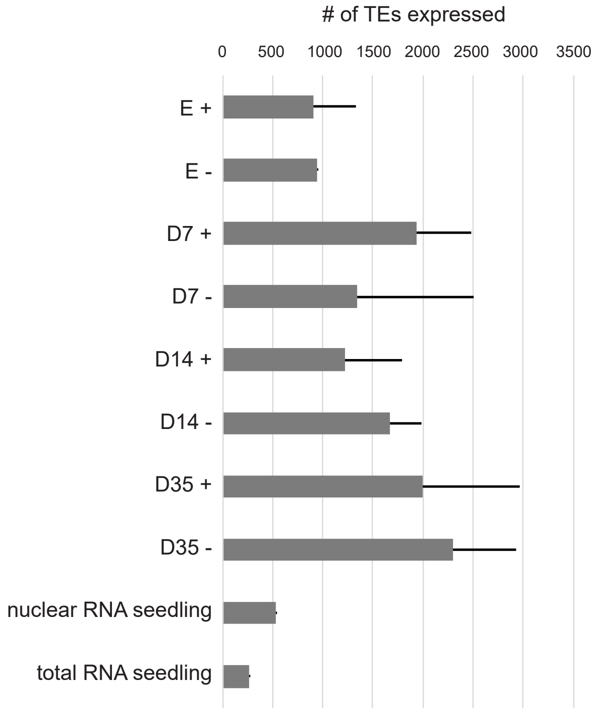


**Appendix Figure S4 | Raw read count numbers of expressed (at least 1 read) TEs per timepoint.** E = nuclei from embryos, D7/14/35 = nuclei from 7/14/35 day-old plants, + = stem cells; - = non-stem cells.

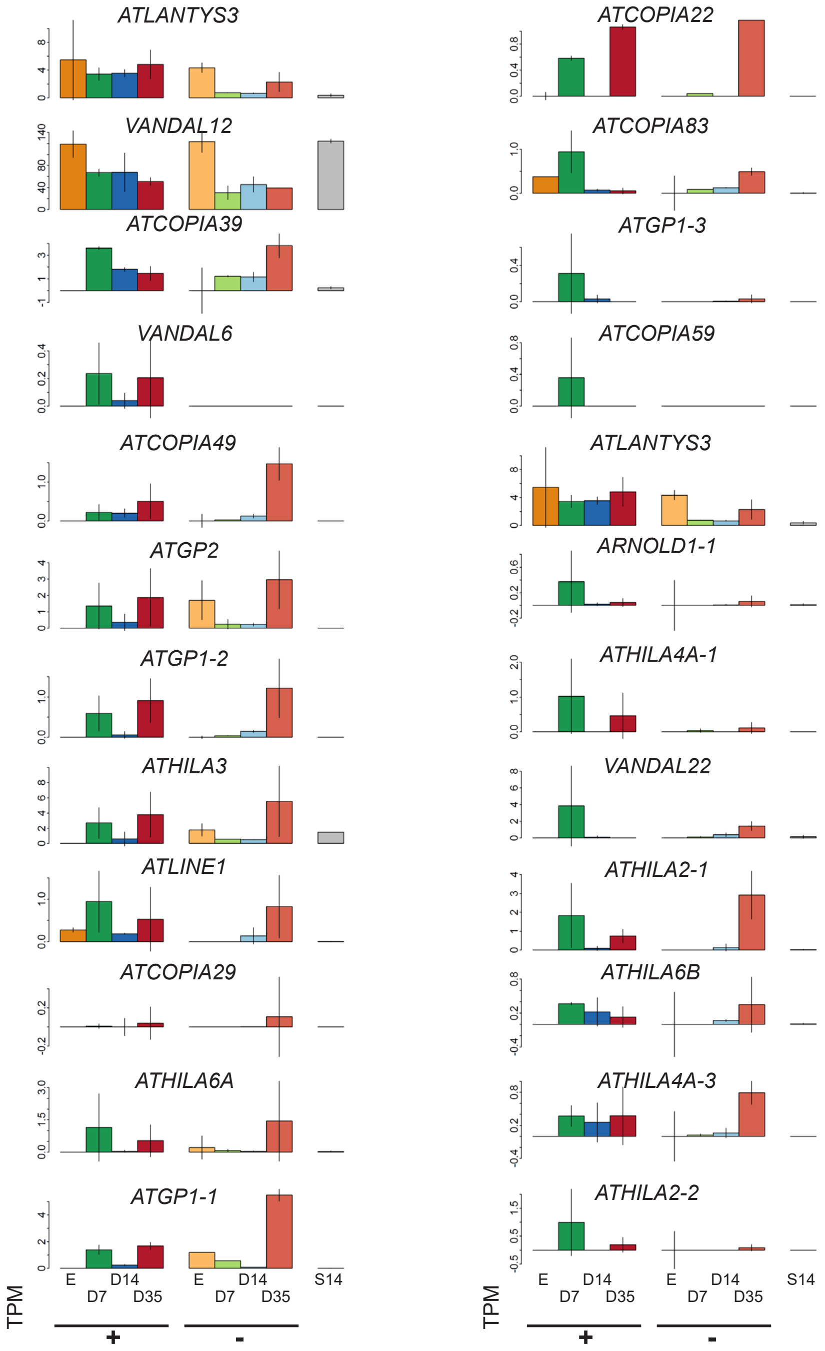


**Appendix Figure S5 | Expression of transposons.**

Example bar plots of TPM values of transposons that are upregulated in D7 SAM stem cells. + = stem cells; - = non stem cells, E = nuclei from embryos, D7/14/35 = nuclei from 7/14/35 day-old plants, S14 = nuclei from 14 d-old above-ground seedlings.

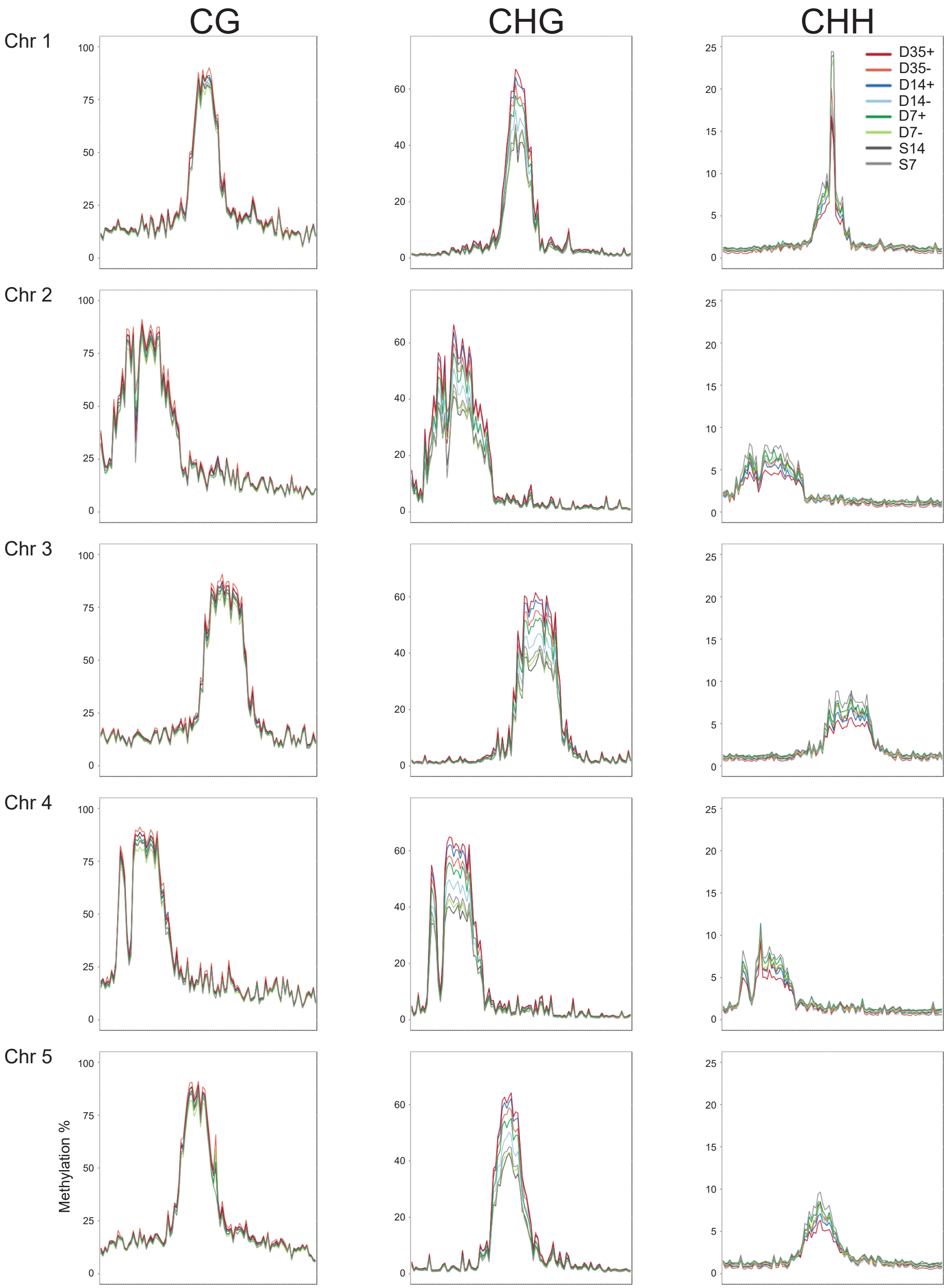


**Appendix Figure S6 | DNA methylation analysis on all five Arabidopsis chromosomes in nuclei of stem (s) and non-stem (n) cells at different developmental stages.** + = stem cells; - = non-stem cells, E = nuclei from embryos, D7/14/35 = nuclei from 7/14/35 day-old plants, S7/14 = nuclei from 7/14 d-old above-ground seedlings.

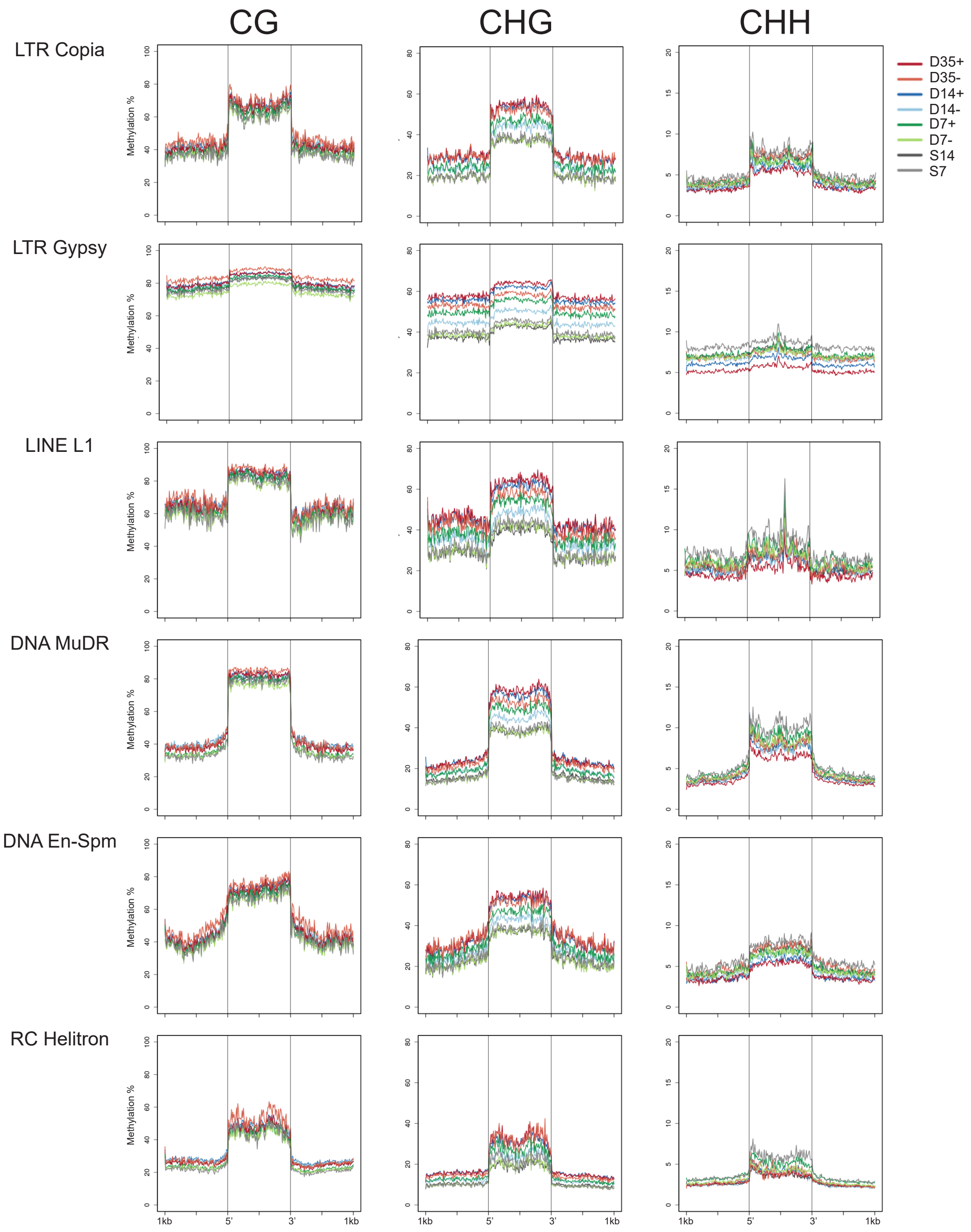


**Appendix Figure S7 | Metaplots of DNA methylation at different TE classes in nuclei of stem (s) and non-stem (n) cells at different developmental stages.** + = stem cells; - = non-stem cells, E = nuclei from embryos, D7/14/35 = nuclei from 7/14/35 day-old plants, S7/14 = nuclei from 7/14 d-old above-ground seedlings.

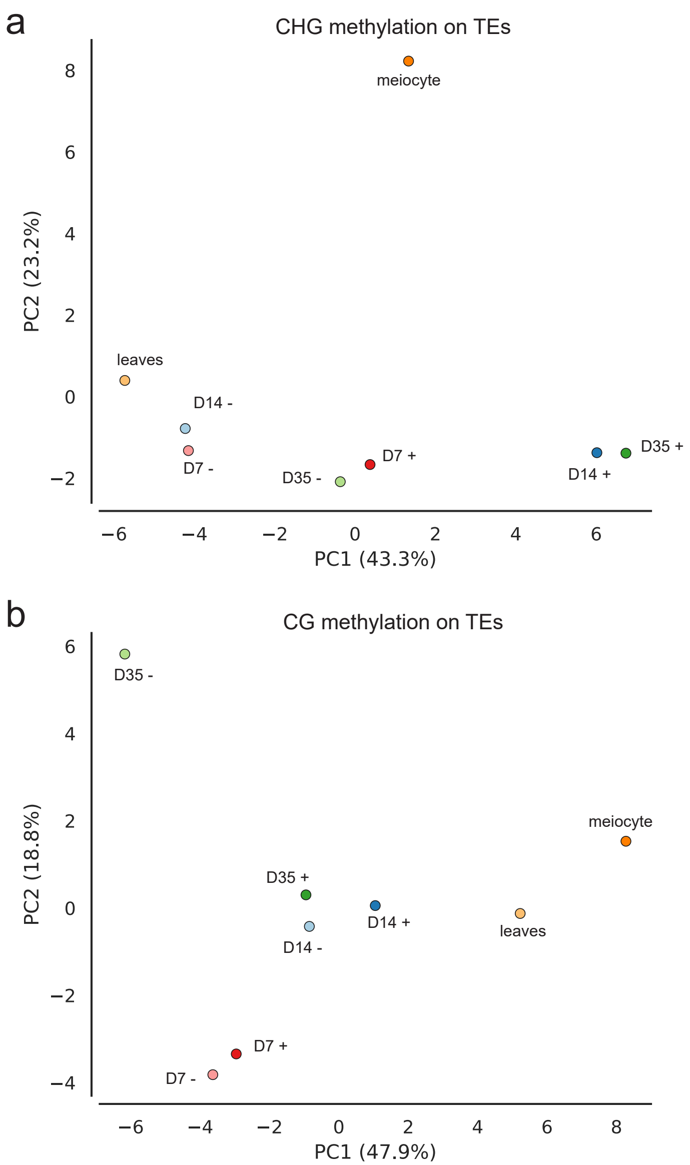


**Appendix Figure S8 |** Principal component analysis for relatedness between (**a**) CHG and (**b**) CG methylation at TEs in meiocytes (data from Walker et al. 2018) and stem (+) and non-stem (-) cell nuclei of 7, 14, or 35 day-old plants (D7/14/35).

**Appendix Table S1**

Examples of FANS data: Number of stem cell nuclei (CLV3+) per total and DAPI events.

| Developmental stage | total number of events | number of DAPI events | number of mCherry events | % (mCherry events/DAPI events)*100 |
|---|---|---|---|---|
| embryo | 1867440 | 48917 | 62 | 0,13 |
| embryo | 1401759 | 50000 | 75 | 0,15 |
| embryo | 1431609 | 50000 | 57 | 0,11 |
| 7d | 100000 | 48818 | 75 | 0,15 |
| 7d | 100000 | 61459 | 159 | 0,26 |
| 7d | 105272 | 36553 | 42 | 0,11 |
| 14d | 100000 | 18255 | 19 | 0,10 |
| 14d | 100000 | 35284 | 26 | 0,07 |
| 14d | 100000 | 35606 | 39 | 0,11 |
| 35d | 100000 | 78945 | 35 | 0,04 |
| 35d | 63518 | 37961 | 20 | 0,05 |
| 35d | 100000 | 80699 | 34 | 0,04 |
