# Supplementary material for: Arabidopsis shoot stem cells display dynamic transcription and DNA methylation patterns
Source: EMBO J. 2020 Aug 20;39(20):e103667. doi: 10.15252/embj.2019103667 (PMC7560203; doi:10.15252/embj.2019103667)
Supplement: Supplementary file 2 — Expanded View Figures PDF [file EMBJ-39-e103667-s002.pdf]

Expanded View Figures

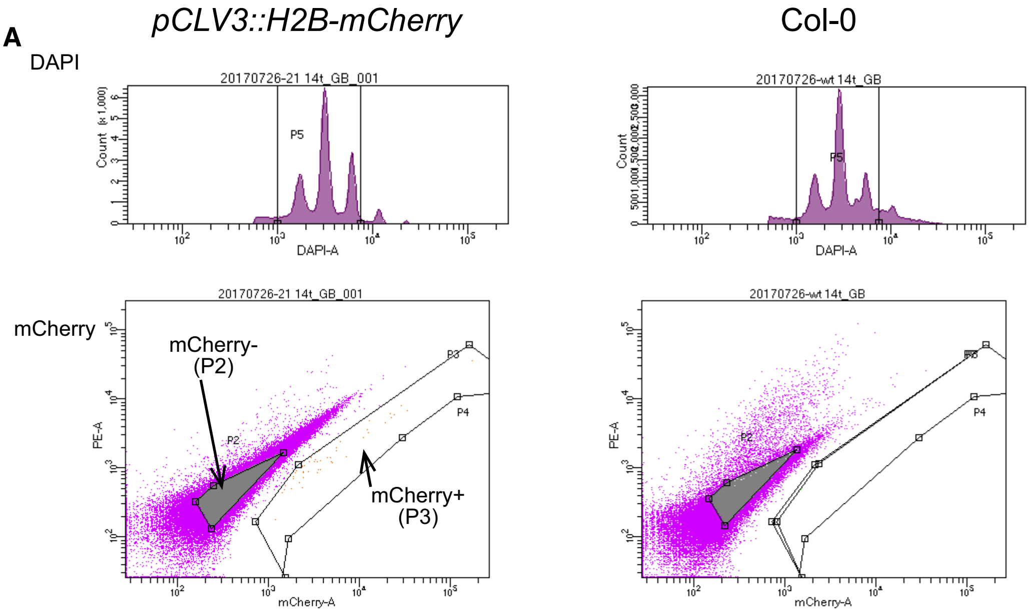

Tube: 21 14t_GB_001

Population	#Events	%Parent	%Total
All Events	2,297,362	####	100.0
P6	2,223,866	96.8	96.8
P1	102,476	4.6	4.5
P5	90,390	88.2	3.9
P2	60,211	66.6	2.6
P4	0	0.0	0.0
P3	49	0.1	0.0

Tube: wt 14t_GB

Population	#Events	%Parent	%Total
All Events	1,232,247	####	100.0
P6	1,213,889	98.5	98.5
P1	57,494	4.7	4.7
P5	46,485	80.9	3.8
P2	19,623	42.2	1.6
P4	0	0.0	0.0
P3	1	0.0	0.0

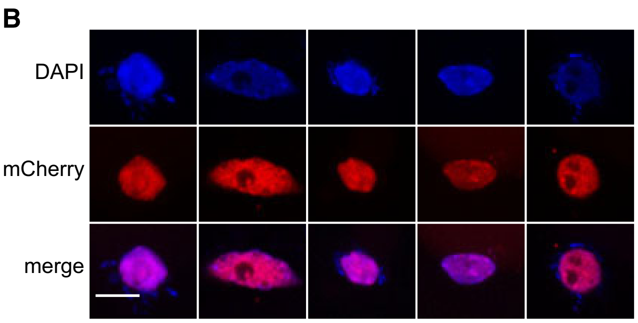

Figure EV1. Isolation of stem cell nuclei and RNA comparison.

A Gating strategy used for FANS of stem cells. Representative FANS plots are shown. Events are gated for DAPI (top row) and next either for mCherry^{+} or mCherry^{-} (bottom row). For numbers, see also Appendix Table S1. Reprinted with permission from Gutzat and Mittelsten Scheid (2020).

B Examples of mCherry-positive nuclei after FANS (scale bar 5 µm).

 © 2020 The Authors

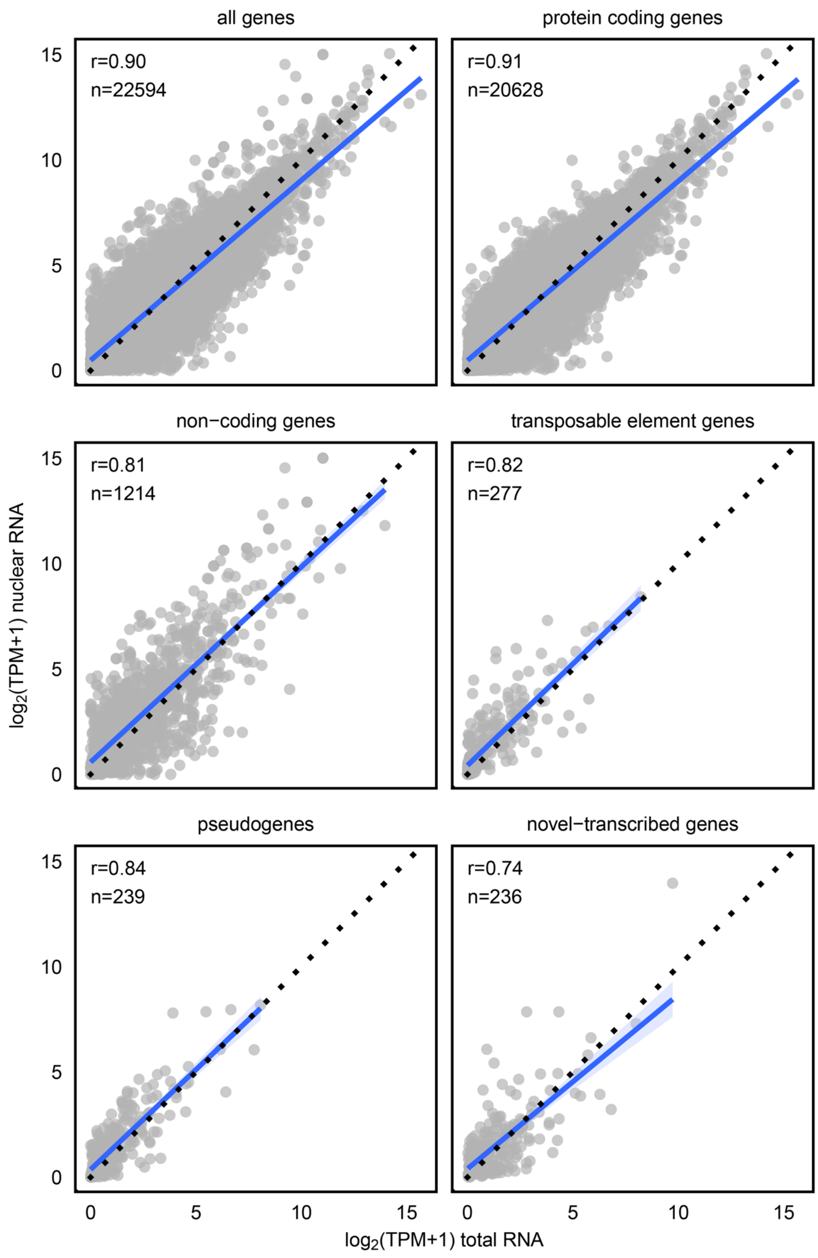

Figure EV2. Correlation of genomic features.

Log_2-normalized TPM values of nuclear and total RNA extracted from 14-day-old seedlings for different genomic features. Only expressed features were used (TPM > 0 in at least one sample). Shown is the number of features and Pearson correlation coefficients.

© 2020 The Authors

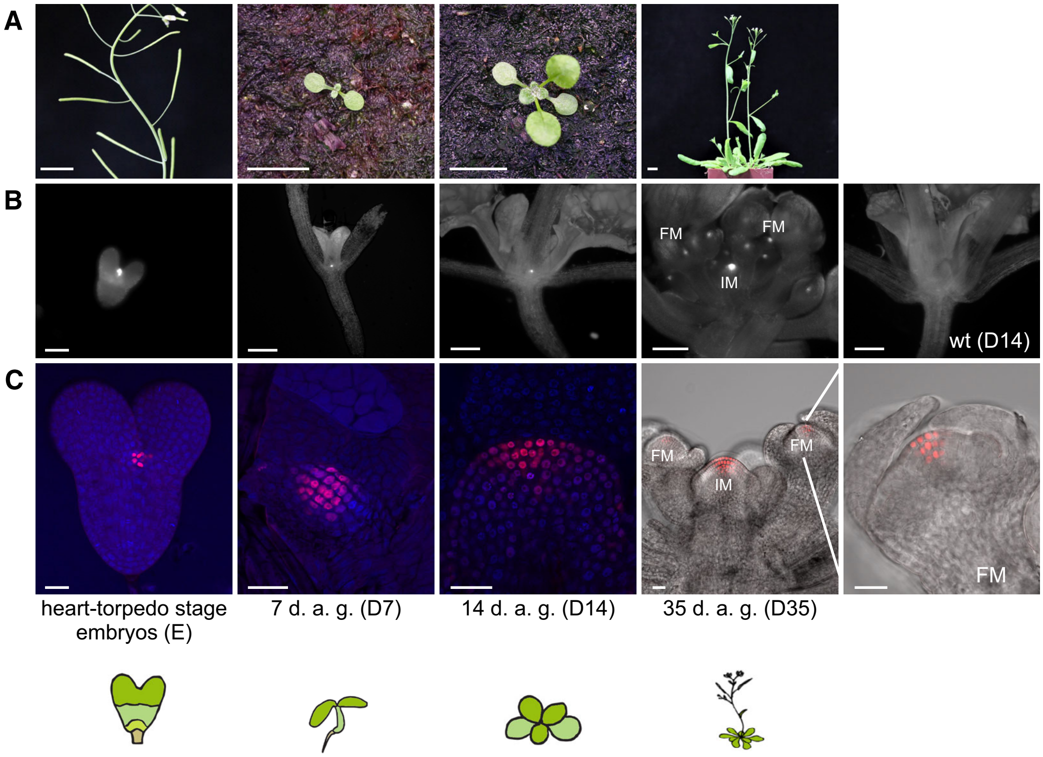

Figure EV3. Growth stages used for genome-wide expression and DNA methylation analysis in stem and non-stem nuclei.

A Developmental stages of representative plants (scale bars 1 cm).

B Wide-field microscopic images with RFP filters.

C LSM pictures of representative plants. For better visualization, DAPI was used as counterstain in E, D7 and D14. FM, floral meristem; IM, inflorescence meristem.

Data information: Scale bars in (B): 60 μm for the embryo; 1 mm for the other three stages. Scale bars in (C): 20 μm.

 © 2020 The Authors
